# Effects of acute unilateral ovariectomy to pre-pubertal rats on steroid hormones secretion and compensatory ovarian responses

**DOI:** 10.1186/1477-7827-9-41

**Published:** 2011-03-30

**Authors:** Leticia Morales-Ledesma, Deyra A Ramírez, Elizabeth Vieyra, Angélica Trujillo, Roberto Chavira, Mario Cárdenas, Roberto Domínguez

**Affiliations:** 1 Biology of Reproduction Research Unit, Physiology of Reproduction Laboratory, Facultad de Estudios Superiores Zaragoza, UNAM, AP 9-020, CP 15000, México DF, México; 2 Escuela de Biología, Benemérita Universidad Autónoma de Puebla, Puebla, México; 3 Instituto Nacional de Ciencias Médicas y Nutrición "Salvador Zubirán", México DF, México

## Abstract

In the present study we analyzed the existence of asymmetry in the secretion of steroid hormones in pre-pubertal female rats treated with unilateral ovariectomy (ULO) or unilateral perforation of the abdominal wall (sham-surgery). Treated rats were sacrificed at different times after surgery. Since sham-surgery had an apparent effect on the age of first vaginal estrous (FVE) and serum levels hormone, the results of the sham surgery groups were used to assess the effects of their respective surgery treatment groups. On the day of FVE, compensatory ovulation (CO) and compensatory ovarian hypertrophy (COH) were similar in animals with ULO, regardless of the ovary remaining *in situ*. In ULO treated animals, progesterone (P4) levels were higher than in animals with sham-surgery one hour after treatment but lower in rats sacrificed at FEV. Left-ULO resulted in lower testosterone (T) concentration 48 and 72 hours after surgery. In rats with Right-ULO lower T concentrations were observed in rats sacrificed one or 72 hours after surgery, and at FVE. ULO (left or right) resulted in lower estradiol (E2) concentrations one or 72 hours after treatment. In rats with Left-ULO, E2 levels were higher 48 hours after surgery and at FVE. Left-ULO resulted in higher levels of follicle stimulating hormone (FSH) five hours after surgery and at FVE. FSH levels were higher in rats with Right-ULO sacrificed on FVE. The present results suggest that in the pre-pubertal rat both ovaries have similar capacities to secrete P4, and that the right ovary has a higher capacity to secrete E2. Taken together, the present results support the idea that the effects of ULO result from the decrease in glandular tissue and changes in the neural information arising from the ovary.

## Background

Unilateral ovariectomy (ULO) is an experimental model used to analyze the existence of functional and physiological asymmetries between the ovaries, including the ability to secrete hormones by each ovary [1]. In non-acute experiments, ULO results in weight increase compensatory ovarian hypertrophy (COH) and in a compensatory ovulation (CO) [2-10].

Compensatory ovarian functions have been explained as resulting from a hormonal imbalance of the hypothalamus-pituitary-ovarian axis caused by the elimination of one source of steroids (i.e. ULO). Following ULO treatment, the pituitary increases the release of follicle stimulating hormone (FSH) [2,3,11-13], which in turn increases the recruitment of small follicles and a decreases in follicular atresia [5,12,14].

The extrinsic innervation of the ovary is involved in COH and CO regulation in the pre-pubertal rat [10,15], the adult rat [2,6,9,16], the pre-pubertal guinea pig [17,18] and in the adult sheep [19].

In the adult rat the acute effects of ULO on progesterone (P_4_), testosterone (T) and estradiol (E_2_) serum levels depends on which ovary remains *in situ *and the day of the estrus cycle when the ovary is removed; suggesting the existence of asymmetry in the ovaries' capacity to secrete steroid hormones [20-23]. In pre-pubertal (28-32 days of age) ULO treated rats, the information arriving to the ovary via the vagus nerve regulates the secretion of ovarian steroid hormones in an asymmetrical way, which depends on the *in situ *ovary and the age of the animal [10].

Several studies show that asymmetries between paired endocrine organs (ovaries, adrenals, thyroid, testis) are related to the regulation exerted by the pituitary trophic hormones [2,6,24,25]. It has been hypothesized that the actions of these hormones are modulated by the glands receiving peripheral innervations, such as the vagus nerve and the superior ovarian nerve [1,20,22,26-28].

To understand the ovaries and pituitary gland compensatory capacity to synthesize hormones, most studies have been performed with adult ULO treated rats.

Because little is known about the ovaries' capacity to secrete steroid hormones at the onset of puberty, the aim of the present study was to evaluate the effects of removing one ovary to pre-pubertal rats on the concentration of steroid hormones and gonadotropins.

## Methods

All experiments were carried out in strict accordance with the Mexican Law of Animal Treatment and Protection Guidelines. The Committee of the Facultad de Estudios Superiores Zaragoza approved the experimental protocols.

Thirty-two day-old female rats of the CII-ZV strain from our own breeding stock were maintained under controlled conditions of light (lights on from 05:00 am to 19:00 pm) and temperature (22 ± 2 °C). Animals were housed in acrylic cages and maintained with free access to rat food (Purina S.A., Mexico) and water *ad libitum*. Animals were kept with their dams until day 21 of age, when they were weaned and placed in group cages (five females and one male per cage). Surgeries were performed under ether anesthesia between 09:00 and 11:00 hours.

This study was designed to assess the effects of unilateral ovariectomy on spontaneous ovulation and the effects on hormones levels. Groups of rats were allotted at random to one of the following treatments:

### A. Effects of unilateral ovariectomy on spontaneous ovulation

#### Untouched controls

Nineteen non-treated rats were sacrificed when they showed their first vaginal estrous (FVE).

#### Sham-surgery or laparotomy (LAP)

Ten rats were treated with a unilateral dorso-lateral incision on the left (L-LAP) side and another ten rats with an incision on the right (R-LAP) side. The incision was performed 2 cm below the last rib; affecting skin, muscle, and peritoneum. No organs were manipulated. The wound was subsequently sealed. When vaginal opening occurred, vaginal smears were obtained daily and the animals were sacrificed on the day of FVE.

#### Unilateral ovariectomy (ULO)

Animals were laparotomized, and the left (L-ULO) [17 rats] or right (R-ULO) [15 rats] ovary was removed. The wound was subsequently sealed. The animals were sacrificed on the day of FVE.

### B. Acute Effects of unilateral ovariectomy on hormones levels

Twenty five groups of eight rats each were assigned to one of the following treatments: control, L-LAP, L-ULO, R-LAP, R-ULO. The animals were sacrificed 30 minutes, one hour, five hours, 48 hours or 72 hours after surgery.

#### Autopsy procedures

The animals were killed by decapitation. The blood of the trunk was collected, allowed to clot at room temperature for 30 minutes and centrifuged at 3,000 RPM for 15 minutes. Serum was stored at -20 °C, until P_4_, T, E_2_, FSH and LH concentrations were measured using radioimmunoassay (RIA). The right and left oviducts were dissected from rats sacrificed on the day of FVE and the number of ova present was counted with a stereoscopic microscope. Ovaries were removed, dissected and weighed on a precision balance.

The CO and COH were calculated as described previously [10]:

CO = [(the number ova shed by the *in situ *ovary - the mean number of ova shed by the respective ovary in the sham surgery group)/the mean number of ova shed by the respective ovary in the sham surgery group] × 100.

COH = [(weight of the *in situ *ovary - the mean weight of the respective ovary from the sham surgery group)/the mean weight of the respective ovary from the sham surgery group] × 100.

#### Hormone measurement

Serum concentrations of E_2 _(pg/ml), T (ng/ml) and P_4 _(ng/ml) were measured using RIA, with kits purchased from Diagnostic Products (Los Angeles, CA, USA). The intra- and inter-assay coefficients of variation were 8.35% and 9.45% for P_4_, 8.12% and 9.28% for E_2_, and 9.65% and 10.2% for T respectively. FSH and LH levels in serum (ng/ml) were measured using the double antibody RIA technique, using reagents and protocols kindly supplied by the NIADDK National Pituitary Program (Bethesda, MD, USA). Intra- and inter-assay variations were in the order of 5.1% and 6.5% for LH, and 4% and 7.9% for FSH. The results are expressed in terms of NIADDK standards RP-2 FSH and LH.

### Statistical analyses

Data on the age of FVE, the number of ova shed, as well as the CO and COH percentages were analyzed using Kruskal-Wallis test, followed by Mann Whitney U-pair-wise comparisons to locate potential differences across treatment groups. Ovulation rates (number of ovulating animals/number of treated animals) were analyzed using Fisher's exact probability test, or the Chi square test. Data on P_4_, T, E_2_, FSH and LH concentrations in serum were analyzed using multivariate analysis of variance (MANOVA), followed by Tukey's test. Differences in hormones serum concentrations between two groups were analyzed with Student's t-test. A *p *value of less than 0.05 was considered significant.

## Results

### Onset of puberty and ovulatory responses

Compared to untreated control rats, sham-surgery treated animals showed a delay in the age of reaching FVE. No changes in ovulation rate or the number of ova shed were observed between control and sham group. Rats with ULO treatment showed similar age of FVE and ovulation rates to sham surgery treated rats; however, the number of ova shed by the *in situ *ovary (left or right) was higher than in rats with sham-surgery (Table 1).

**Table 1:** Ovulatory response in rats with unilateral ovariectomy performed at 32 days of age.

Groups	n	FVE (days)	Number of ova shed per ovary		Ovulation rate
			Left	Right	
**Control**	19	34.9 ± 0.5	4.2 ± 0.4	5.0 ± 0.5	13/19
**L-LAP**	10	38.1 ± 0.9*	5.1 ± 1.2	4.7 ± 0.5	8/10
**L-ULO**	17	39.5 ± 0.6	-	8.5 ± 1.0**	9/17
**R-LAP**	10	40.2 ± 1.2*	4.1 ± 0.7	4.8 ± 0.4	9/10
**R-ULO**	15	39.1 ± 0.9	7.7 ± 1.2**	-	9/15

Age of first vaginal estrus (FVE), number of ova shed (Mean ± S.E.M.) and ovulation rate. Data is presented for control animals, left (L-LAP) and right (R-LAP) laparotomy, left (L-ULO) and right (R-ULO) unilateral ovariectomy at 32 days of age. Animals were sacrificed at FVE.

*p < 0.05 *vs*. control group (Kruskal-Wallis test followed by Mann-Whitney U-test).

**p < 0.05 *vs*. its respective sham-surgery group (Mann-Whitney U-test).

### Effects of LAP on P_4_, T and E_2 _serum levels

**Figure 1:**
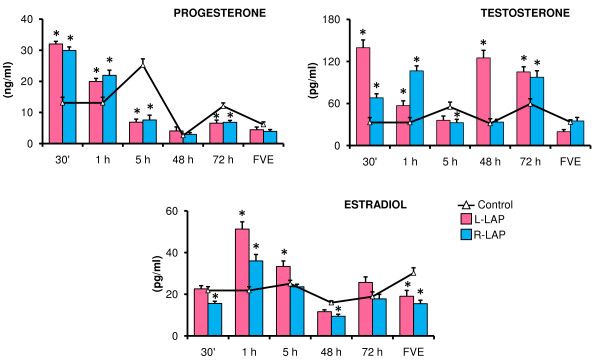
**Steroid hormones serum levels in laparotomized (LAP) rats performed at 32 days of age**. Mean ± S.E.M. of steroid hormones levels in serum. Control, left (L-LAP) or right (R-LAP) laparotomized rats. Animals were sacrificed at various times after surgery *p < 0.05 *vs*. control group (MANOVA followed by a Tukey's test).

In LAP (left or right) treated rats, P_4 _levels were higher in animals sacrificed either 30 minutes or one hour after surgery, but lower in animals sacrificed five or 72 hours after surgery (Figure 1).

L-LAP and R-LAP effects on T and E_2 _levels varied with the time elapsed between surgery and autopsy (Figure 1). Compared to control animals, the effects of left and right LAP treatment on T levels depended on the time between surgery and autopsy: T levels were higher in rats with LAP treatment sacrificed 30 minutes, one hour or 72 hours after surgery. These changes depended on the side of the peritoneum that was perforated (Figure 1).

In both L-LAP and R-LAP treatment groups, E_2 _levels were different than in control groups and these differences were associated to the side (left or right) the surgery was performed and on the time elapsed between surgery and autopsy. E_2 _serum levels were higher in L-LAP treated rats sacrificed one or five hours after treatment, and lower in animals sacrificed at FVE. Compared to the control group, rats in the R-LAP group sacrificed one hour after surgery had higher E_2 _serum levels, and lower E_2 _levels in rats sacrificed 30 minutes or 48 hours after surgery or at FVE (Figure 1).

### Effects of LAP on FSH and LH serum levels

**Figure 2:**
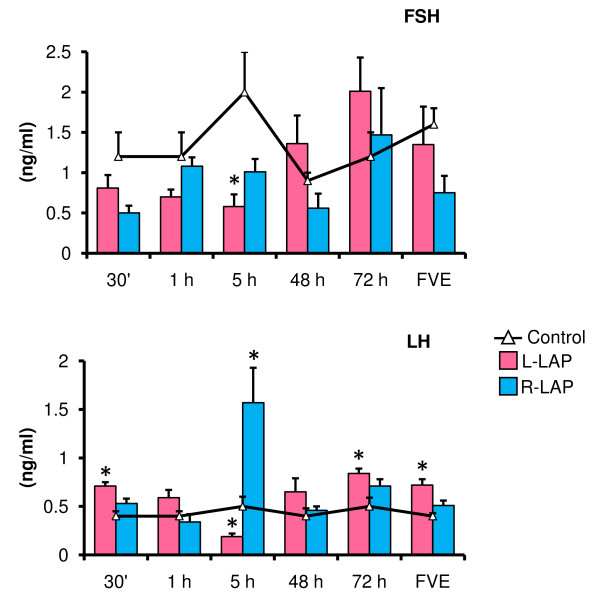
**FSH and LH serum levels in laparotomized (LAP) rats performed at 32 days of age**. Mean ± S.E.M. of FSH and LH levels in serum. Control, left (L-LAP) or right (R-LAP) laparotomized rats. Animals were sacrificed at various times after surgery. *p < 0.05 *vs*. control group (MANOVA followed by a Tukey's test).

Compared to the control group, L-LAP treated animals sacrificed five hours after surgery had lower FSH levels (Figure 2). The effects of LAP on LH depended on the side LAP surgery was performed. Compared to their respective control group, L-LAP resulted in higher LH levels in animals sacrificed at 30 minutes, 72 hours or at FVE. In R-LAP treated rats, higher LH levels were observed in animals sacrificed five hours after surgery (Figure 2).

### CO and COH

Animals with ULO (the left or right ovary *in situ*) sacrificed on FVE had similar CO (left ovary *in situ*142.1 ± 13.9% *vs*. right ovary *in situ *129.3 ± 7.9% NS). COH occurred 48 hours after ULO treatment and was similar for both ovaries (between 35 and 48%).

### Effects of ULO on P_4_, T, and E_2 _serum levels

**Figure 3:**
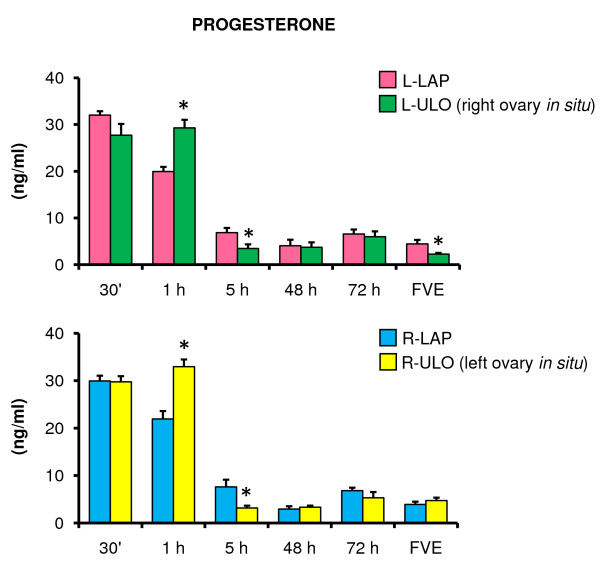
**Progesterone serum levels in rats with unilateral ovariectomy performed at 32 days of age**. Mean ± S.E.M. of progesterone levels in serum. Left (L-LAP) or right (R-LAP) laparotomy, left (L-ULO) or right (R-ULO) unilateral ovariectomy rats. Animals were sacrificed at various times after surgery. *p < 0.05 *vs*. its respective sham-surgery group (Student's t-test).

Compared to LAP treated animals, the P_4 _concentrations in L-ULO and R-ULO treated animals were higher in the group sacrificed one hour after surgery, and lower in the rats sacrificed five hours after treatment. L-ULO resulted in lower P_4 _concentration in animals sacrificed at FVE (Figure 3).

In animals sacrificed at FVE, rats with the right ovary *in situ *had lower P_4 _concentrations than rats with the left ovary *in situ *(right ovary *in situ *2.2 ± 0.2 *vs*. left ovary *in situ *4.7 ± 0.5 ng/ml, p < 0.05)

**Figure 4:**
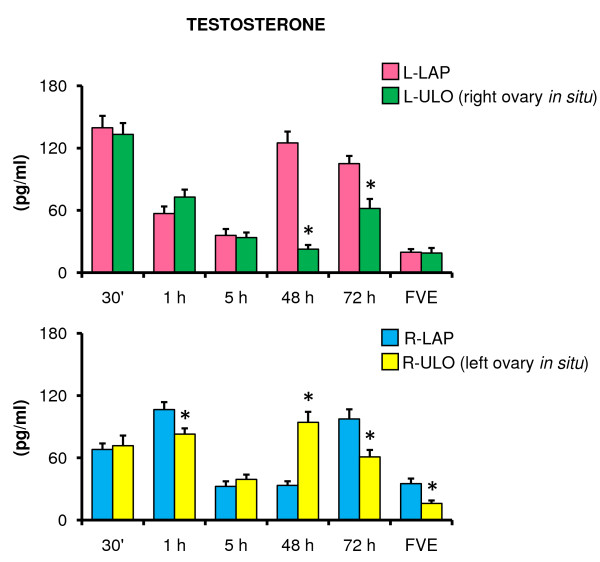
**Testosterone serum levels in rats with unilateral ovariectomy performed at 32 days of age**. Mean ± S.E.M. of testosterone levels in serum. Left (L-LAP) or right (R-LAP) laparotomy, left (L-ULO) or right (R-ULO) unilateral ovariectomy rats. Animals were sacrificed at various times after surgery. *p < 0.05 *vs*. its respective sham-surgery group (Student's t-test).

Compared to their corresponding LAP treatment group, removing the left ovary resulted in lower T concentrations in animals sacrificed 48 or 72 hours after surgery. A similar result was observed in rats with R-ULO sacrificed one or 72 hours after surgery, and those sacrificed at FVE. R-ULO animals sacrificed 48 hours after surgery showed significantly higher T concentrations than the sham-surgery group (Figure 4).

Thirty minutes after ULO the right ovary's capacity to secrete T was higher than the left ovary (133.2 ± 10.8 *vs*. 71.6 ± 9.8 pg/ml, p < 0.05). In animals sacrificed 48 hours after surgery the left ovary secreted significantly more T than the right one (94.2 ± 10.1 *vs*. 22.6 ± 3.1 pg/ml, p < 0.05).

**Figure 5:**
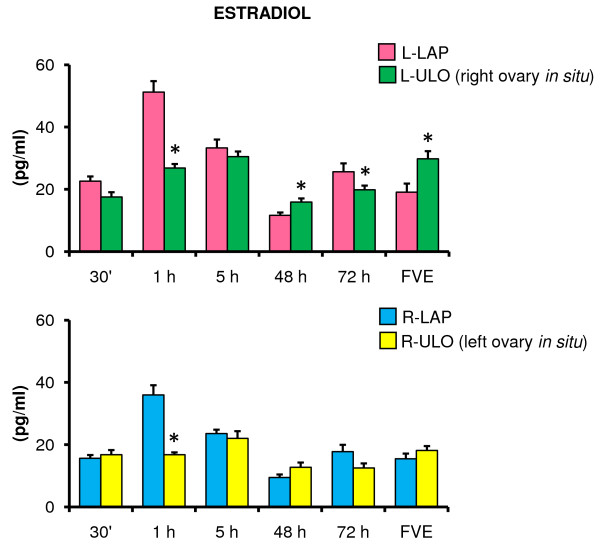
**Estradiol serum levels in rats with unilateral ovariectomy performed at 32 days of age**. Mean ± S.E.M. of estradiol levels in serum. Left (L-LAP) or right (R-LAP) laparotomy, left (L-ULO) or right (R-ULO) unilateral ovariectomy rats. Animals were sacrificed at various times after surgery. *p < 0.05 *vs*. its respective sham-surgery group (Student's t-test).

Rats with ULO (left or right) treatment had lower E_2 _concentrations than LAP treated animals sacrificed one or 72 hours after treatment. L-ULO treated animals had higher E_2 _levels 48 hours after surgery and at FVE (Figure 5).

In right ovary appears to have a greater capacity to secrete E_2 _than the left ovary at one, five and 72 hours after ULO treatment and at FVE (1 h 26.8 ± 1.3 *vs*. 16.7 ± 0.7; 5 h 30.5 ± 1.6 *vs*. 22.0 ± 2.3; 72 h 19.8 ± 1.*3 vs*. 12.5 ± 1.4; FVE 29.7 ± 2.4 *vs*. 18.1 ± 1.4 pg/ml, p < 0.05).

### Effects of ULO on FSH and LH serum levels

**Figure 6:**
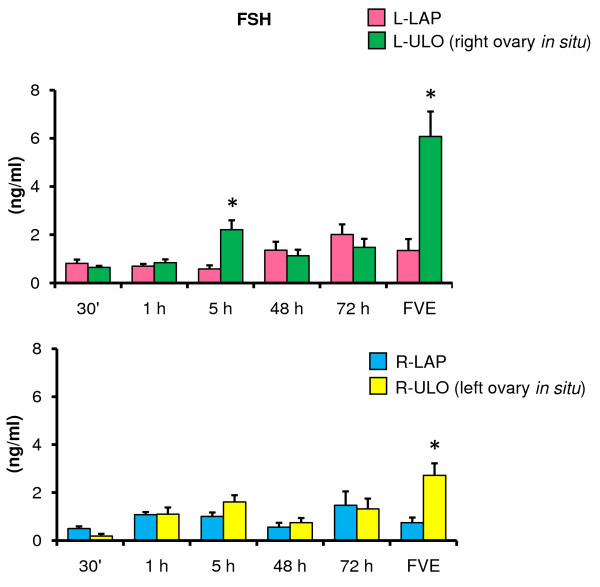
**FSH serum levels in rats with unilateral ovariectomy performed at 32 days of age**. Mean ± S.E.M. of FSH levels in serum. Left (L-LAP) or right (R-LAP) laparotomy, left (L-ULO) or right (R-ULO) unilateral ovariectomy. Animals were sacrificed at various times after surgery. *p < 0.05 *vs*. its respective sham-surgery group (Student's t-test).

Compared to LAP animals, L-ULO treatment resulted in higher FSH levels five hours after surgery and at FVE. In R-ULO treated animals FSH increased at FVE (Figure 6). In rats with the right ovary *in situ *(L-ULO), FSH levels were higher than in animals with the left ovary *in situ *(FVE 6.0 ± 1.0 *vs*. 2.7 ± 0.5 ng/ml, p < 0.05).

**Figure 7:**
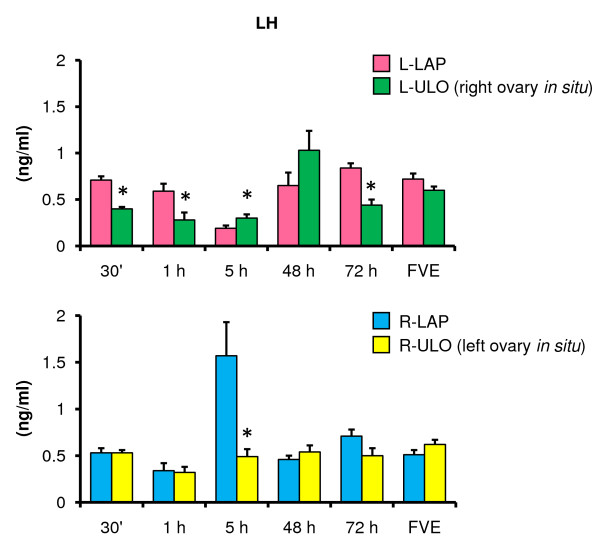
**LH serum levels in rats with unilateral ovariectomy performed at 32 days of age**. Mean ± S.E.M. of LH levels in serum. Left (L-LAP) or right (R-LAP) laparotomy, left (L-ULO) or right (R-ULO) unilateral ovariectomy rats. Animals were sacrificed at various times after surgery. *p < 0.05 *vs*. its respective sham-surgery group (Student's t-test).

Compared to their corresponding LAP treatment group, LH levels in L-ULO treated animals were lower at 30 minutes, one and 72 hours after surgery. LH levels were higher in R-ULO animals sacrificed five hours after surgery (Figure 7).

## Discussion

Present results suggest that in the pre-pubertal rat, the left and right ovaries have similar capacities to secrete P_4_, but the right ovary has a higher capacity to secrete E_2_.

COH is similar in pre-pubertal and adult rats with ULO treated on diestrus 1 or diestrus 2 [6,13], suggesting that the mechanisms regulating follicular growth are similar. The capacity for CO in the adult rat is asymmetric, the left ovary has a higher CO capacity than the right one, and varies depending to the day of the cycle when surgery was performed [1,13]. Present results show that in pre-pubertal rats such asymmetry does not occur, suggesting that in pre-pubertal rats the mechanisms regulating CO (follicular growth and differentiation) are similar to those occurring in rats at diestrus [1].

Based on the effects of unilaterally stimulating the hind-paw or abdomen of female rats on ovarian sympathetic nerve activity, Uchida et al. [29] suggested the existence of a supra-spinal reflex pathway that is independent of the laterality of the stimulus applied at hind-paw, and a spinal segmental reflex pathway that depends on the laterality of the stimulus applied at the abdomen. The acute effects of unilateral laparotomy on P_4_, T, and E_2 _levels depend on the side and the day of the estrous cycle when surgery was performed, supporting the idea of a spinal segmental reflex pathway that varies during the estrous cycle [20-23]. The neural information arising from the dorsal and ventral peritoneum plays different roles on the mechanism regulating ovarian steroid hormone secretion [30]. Similar results were observed in the present study, where T and E_2 _levels changed following laparotomy and depended on the side the incision was made and the time elapsed between surgery and autopsy. Unilateral LAP did not elicit an apparent asymmetry in P_4 _levels, suggesting that in the pre-pubertal rat the mechanisms regulating P_4 _secretion are not the same as in adult animals.

In the adult cyclic rat, ULO leads to a functional compensation that is observed at 15 days and thereafter after surgery [31]. Such results were explained by the drop in P_4 _or E_2 _levels followed by an increase in FSH secretion [32,33]. Injecting P_4 _or E_2 _to ULO rats did not modify the increase in FSH levels, while injecting bovine follicular fluid (BFF) blocked the rise in FSH levels that follow the removal of one [3,34]. Welschen et al. [34] showed that after ULO treatment, FSH levels increased over a four to 28 h period after surgery; P_4 _levels dropped within two to 24 h; and no significant changes occurred in LH and E_2 _levels. These results suggest that in the rat, after ULO treatment the higher FSH levels are induced by a transient decrease in peripheral levels of a non-steroidal inhibin-like ovarian factor [34].

The acute effects of ULO on P_4 _serum levels depends on which ovary remains *in situ *and on the day of the estrous cycle when surgery is performed [20,21]. Present results show that in the late phase of the rat's pre-pubertal development the left and right ovaries have similar capacities for P_4 _synthesis.

In present study, the difference in P_4 _synthesis between the left and right ovary at the FVE, does not depend on the number of corpora lutea since ovulation rates were similar in both ovaries. Such disparities in the P_4 _levels could be related to the adrenal innervations arising from the celiac-superior mesenteric ganglia (CSMG). The asymmetry in the neuronal activity observed in the CSMG of the adult rat [35] is not present in 28 days old pre-pubertal rats [36].

Present results show that the asymmetry in the ovaries' capacity to secrete T and E_2 _is different between the adult and the pre-pubertal rat. In the pre-pubertal rat the right ovary secretes more E_2_, while in the adult rat the left ovary secretes more P_4_, T and E_2 _[20-23].

Furthermore, according to Kawakami et al. [37,38] the electric stimulation of the medio-basal prechiasmathic area and the ventromedial hypothalamus to hypophysectomized and adrenalectomized rats provoked the release of P_4 _and E_2_, with no apparent changes in GnRH and gonadotropins levels or ovarian blood flow, suggesting a direct neural control of the ovarian steroidogenesis. Gerendai et al. [39] show the existence of a direct neural pathway between the ovary and the CNS, and postulated that this neural connection exerts a pituitary-independent, purely neural regulatory action on the ovary.

Aside from the hormonal participation in the compensatory responses observed after ULO, there are evidences suggesting that ovarian innervations arriving to the ovaries through the vagus nerves and the superior ovarian nerve (SON) play a role in compensatory ovarian responses [2,6-8,10,13,15,16]. The compensatory effects following ULO depends on the *in situ *ovary and the day of the estrous cycle when ULO was performed [21-23]. Then, following ULO treatment, the compensatory process by the ovaries would depend on changes in the hormonal and neural signals arising and arriving to the ovaries, which appear to be different for the right and left ovary [1]. The neural information arriving and leaving the ovaries through the vagus nerve and the SON plays a role in regulating steroid hormone secretion by the ovaries in a lateralized way [10,15].

The ovarian innervations modulate ovarian responses to gonadotropins [1]. In consequence, the asymmetric ability of the ovaries to secrete steroid hormones could be related to the amount of neural connections between the hypothalamus and the ovaries [35,36,40,41]. Kagitani et al. [42] suggest that the autonomic nerves that reach the ovary via the SON play an inhibitory role in the secretion of ovarian E_2_, while other studies suggest that the vagus nerve and SON innervations to the ovary participate in the regulation of hormone secretion and that hormone secretion varies along the estrous cycle [21-23].

Then, the acute changes in ovarian hormone levels following ULO could be explained by the lack of neural information arising from the extirpated ovary, which plays a role regulating ovarian functions [1]. The sub-acute modifications in ovarian hormone release induced by ULO would depend on changes in gonadotropins levels and the re-arrangement of neural factors that regulate ovarian steroidogenesis on the remaining ovary *in situ*.

## Conclusions

The present results suggest that in the pre-pubertal rat both ovaries have similar capacities to secrete P_4_, and that the right ovary has a higher capacity to secrete E_2_.

Taken together, present results support the idea that the effects of ULO result from the decrease in glandular tissue and changes in the neural information arising from the ovary

## Competing interests

The authors declare that they have no competing interests.

## Authors' contributions

LM and RD planned the experiments. LM, DAR, EV, AT and RD devised the study and participated in the discussion of the results. RC and MC participated in performing the RIA's to measure the different hormones levels. All authors read and approved the final manuscript.
